# Emission of Toxic HCN During NO_*x*_ Removal by Ammonia SCR in the Exhaust of Lean‐Burn Natural Gas Engines

**DOI:** 10.1002/anie.202003670

**Published:** 2020-07-01

**Authors:** Deniz Zengel, Pirmin Koch, Bentolhoda Torkashvand, Jan‐Dierk Grunwaldt, Maria Casapu, Olaf Deutschmann

**Affiliations:** ^1^ Institute for Chemical Technology and Polymer Chemistry Karlsruhe Institute of Technology Engesserstr. 20 76131 Karlsruhe Germany

**Keywords:** ammonia, formaldehyde, hydrogen cyanide, selective catalytic reduction

## Abstract

Reducing greenhouse gas and pollutant emissions is one of the most stringent priorities of our society to minimize their dramatic effects on health and environment. Natural gas (NG) engines, in particular at lean conditions, emit less CO_2_ in comparison to combustion engines operated with liquid fuels but NG engines still require emission control devices for NO_*x*_ removal. Using state‐of‐the‐art technologies for selective catalytic reduction (SCR) of NO_*x*_ with NH_3_, we evaluated the interplay of the reducing agent NH_3_ and formaldehyde, which is always present in the exhaust of NG engines. Our results show that a significant amount of highly toxic hydrogen cyanide (HCN) is formed. All catalysts tested partially convert formaldehyde to HCOOH and CO. Additionally, they form secondary emissions of HCN due to catalytic reactions of formaldehyde and its oxidation intermediates with NH_3_. With the present components of the exhaust gas aftertreatment system the HCN emissions are not efficiently converted to non‐polluting gases. The development of more advanced catalyst formulations with improved oxidation activity is mandatory to solve this novel critical issue.

## Graphical abstract

**HCN formation over common SCR catalysts**: Natural gas engines emit less CO_2_ in comparison to common liquid fuel combustion engines and are therefore an attractive alternative. Nevertheless, they still require emission control devices for NO_*x*_ removal. As HCHO is present in the exhaust, this study was focused on the interplay of HCHO and other compounds of an SCR gas mixture and revealed the formation of HCN over a broad variety of common NH_3_‐SCR catalysts.

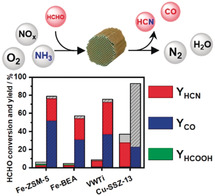

The growing global awareness towards climate change has led to the introduction of alternative fuels able to reduce the net greenhouse gas emissions. In addition to fuel‐ethanol blends, liquid petroleum gas, and biodiesel, natural gas has shown to be one of the most promising candidates for reducing up to 20 % the anthropogenic CO_2_ emissions per produced energy unit.^1^ This benefit is due to the high H/C ratio of methane, the major component of natural gas (up to 97 %). The growing interest in natural gas as fuel is also boosted by the possibility to produce methane from CO_2_‐neutral sources such as biomass and even more important from wind and solar derived electricity by the power to gas (PtG) technology, which is a combination of electrolysis of steam and subsequent methanation.^2^ In contrast to diesel and gasoline powered engines, the combustion process of methane is almost free of particulate matter (PM) emissions due to the absence of long hydrocarbon chains in the fuel, which is regarded as a positive aspect particularly for decreasing local air pollution. As a consequence, the number of natural gas fueled vehicles is expected to increase,^3^ as also predicted by the energy transition trends. However, natural gas engines still require a catalytic exhaust‐gas aftertreatment system.^4^ In addition to the ultimate chemical products of complete combustion, CO_2_ and water, harmful gases such as nitrogen oxides (NO_*x*_), carbon monoxide (CO), volatile organic compounds (VOC), light hydrocarbons including unburnt methane (CH_4_) as well as carbonyl intermediates formed during partial oxidation of methane need to be removed.^5^ CO, NO_*x*_, and hydrocarbons (in total) belong to the group of regulated emissions. Regulations on specific hydrocarbon species such as formaldehyde (HCHO) and non‐methane hydrocarbons (NMHC) have started to be introduced for some applications in various regions of the world. Emission standards will continuously advance and also include other combustion products that are known for their toxicity and greenhouse impact.^6^

Depending on the air‐fuel ratio, a natural gas (NG) combustion engine can be operated under stoichiometric and lean (excess of oxygen) conditions, with the last one showing an improved thermal efficiency and therefore less fuel consumption. Even though the concentration of CO, HC and especially NO_*x*_ emissions in the exhaust stream is higher for the stoichiometric engines, the removal of all three pollutant classes can be efficiently achieved over a conventional three‐way catalyst. To comply with the tightened NO_*x*_ emission limits, the exhaust aftertreatment system of the lean‐burn NG engines requires the application of a NO_*x*_ reduction catalyst.^4^ In this respect, the selective catalytic reduction (SCR) of NO_*x*_ with ammonia is currently the most efficient aftertreatment technology, using ion‐exchanged zeolites or vanadium‐based catalyst formulations. The NO_*x*_‐removal catalyst is typically exposed to a lean gas mixture containing nitrogen oxides and small amounts of unreacted components (methane slip) or oxidation by‐products as pollutants. Among them, formaldehyde emissions formed due to incomplete combustion and partial oxidation of methane in the hot exhaust stream require special consideration, as formaldehyde is known as a potential carcinogenic compound regulated since 2014.5b, 7 As shown by recent studies,^8^ fresh noble metal‐based oxidation catalysts are able to significantly convert formaldehyde. However, when using more complex gas mixtures8a, 8b or upon catalyst ageing8a, 9 (i.e., SO_2_ poisoning or field aging) the activity significantly decreases, particularly at low temperatures. Furthermore, complete conversion is virtually impossible to achieve at high gas hourly space velocity with a typical catalyst length due to the low diffusion rate of formaldehyde from the gas phase to the catalyst surface, especially at low concentrations.^9^, ^10^

When evaluating the impact of formaldehyde presence on the NO_*x*_ removal performance of a series of conventionally applied SCR catalysts for the exhaust aftertreatment of lean‐burn NG engines, we identified the formation of the highly toxic hydrogen cyanide (HCN) over the catalyst bed during the NH_3_‐SCR process. It is well known that the exposure to over 300 ppm HCN in air kills within several minutes and thirty minutes exposure to 135 ppm HCN in air can be lethal.^11^ Up to now, HCN emissions have been encountered predominantly in mining industry, metallurgical plants and biomass burning.^12^ At much lower concentration, hydrogen cyanide was also found in the exhaust of gasoline and diesel vehicles, directly formed during fossil fuel combustion, SCR of NO_*x*_ with hydrocarbons,^13^ dehydration of methanamide (intermediate/side‐product during NH_3_ generation from ammonium formate) over NH_3_‐SCR catalysts^14^ or for malfunctioning three‐way catalysts.^15^ However, to the best of our knowledge, the formation of HCN has never been reported for natural gas engines, especially as a result of a catalytic reaction between formaldehyde and ammonia.

In order to obtain a complete overview on the commercially available NH_3_‐SCR catalyst technologies, four different catalysts have been used in our study: 1.3 % Fe‐ZSM‐5, 1.4 % Fe‐BEA, 1.7 % Cu‐SSZ‐13 and 2 % V_2_O_5_, 9 % WO_3_/TiO_2_. The catalytic tests were performed with catalyst coated honeycombs at typical technical conditions, i.e., a gas hourly space velocity (GHSV) of 100 000 h^−1^ using a synthetic SCR gas mixture of 0/175/350 ppm NO, 0/175 ppm NO_2_, 0/350 ppm NH_3_, 0/80 ppm HCHO, 12 % H_2_O, 10 % O_2_ and N_2_ balance. This formaldehyde concentration of 80 ppm was selected based on direct engine measurements^16^ and also to ensure a high accuracy of the measured values for the different gaseous products of formaldehyde oxidation. More details on the catalyst preparation and testing procedure are provided in the Supporting Information. The results depicted in Figure 1 illustrate the impact of formaldehyde presence in the gas stream on the standard NO_*x*_ conversion for the Fe‐ZSM‐5 catalyst. A slightly increased NH_3_ consumption is observed above 250 °C simultaneously with the decrease of NO_*x*_ reduction (Figure 1 A). During this process, HCHO is gradually converted to CO and HCN, reaching 90 % conversion at 550 °C (Figure 1 B). The selectivity towards hydrogen cyanide increases with temperature up to 50 % at 400 °C, followed by a decrease to only 20 % at 550 °C. The oxidation process over the Fe‐ZSM‐5 catalyst leads also to high CO emissions, with 75 % selectivity at the highest investigated temperature. In addition, small traces of formic acid were measured at low temperatures (Figure 1 B). Considering that at low temperatures NH_3_ is known to directly react with aldehydes to form amines,^17^ we also cannot exclude the formation of such compounds below 300 °C,^18^ which would close the carbon balance at these temperatures. This reaction is also suggested by the slightly higher HCHO conversion at 150 °C vs. 200 °C (Figure S6 in the Supporting Information). Moreover, the formation of CO_2_ in this temperature range is unlikely since the CO conversion onset on Fe‐ZSM‐5 is only observed above 350 °C (Figure S8).

**Figure 1:**
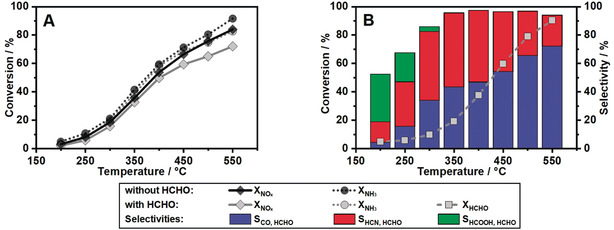
(A) Comparison of NO_*x*_ and NH_3_ conversion over Fe‐ZSM‐5 during standard SCR (350 ppm NO, 350 ppm NH_3_, 12 % H_2_O, 10 % O_2_ in N_2_) with and without 80 ppm HCHO. (B) HCHO conversion and product selectivity towards CO, HCN, and HCOOH.

As already indicated by the NH_3_ overconsumption relative to the NO conversion (Figure 1 A), the formation of HCN seems to be directly linked to a reaction between HCHO or its oxidation intermediates/by‐products and NH_3_. Since under standard SCR conditions no gas phase reactions leading to hydrogen cyanide could be observed during empty reactor tests (Figure S3), the HCN production obviously is a consequence of HCHO reactions on the SCR catalyst. In contrast to previous studies in literature, which reported the formation of HCN by the reduction of NO with CO15a or other hydrocarbons,13b, 13c, 13d our results demonstrate a similar selectivity trend towards HCN formation but in this case due to the reaction between HCHO and NH_3_ (Figure 2 A vs. Figure 2 B). Thus, by comparing the NO oxidation (Figure 2 A) in presence and absence of HCHO it could be observed that the conversion of HCHO is competing with the oxidation of NO for active sites, and therefore results in a decreased NO oxidation activity. However, no emissions of HCN could be measured. Indeed, only significant CO emissions and HCOOH traces were detected during formaldehyde‐only or formaldehyde and NO oxidation on Fe‐ZSM‐5 (Figure 2 A, Figure S5). Also, for a stream containing CO and the standard SCR gas mixture, no secondary emissions were observed (Figure S8), suggesting that not CO but an oxidation intermediate of HCHO is responsible for the formation of HCN.

**Figure 2:**
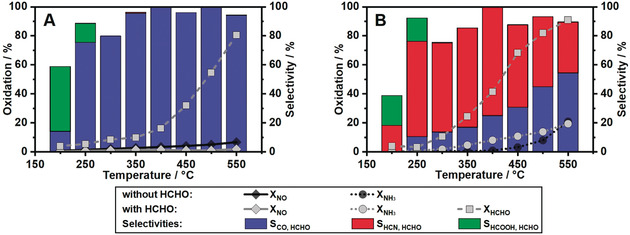
Simultaneous oxidation of (A) NO and HCHO or (B) NH_3_ and HCHO over Fe‐ZSM‐5. Comparison of conversion and product selectivity with and without HCHO in a gas mixture consisting of 350 ppm NO/NH_3_, 0–80 ppm HCHO, 12 % H_2_O, 10 % O_2_ in N_2_.

In case of NH_3_ oxidation (Figure 2 B) in presence of HCHO, the oxidation of NH_3_ is enhanced up to 550 °C. Simultaneously, the conversion of formaldehyde increased compared to the NO oxidation profile in the same temperature window. This increment in HCHO conversion could be directly linked to the formation of HCN. Hence, a possible mechanism for hydrogen cyanide formation from HCHO during NH_3_‐SCR could involve the oxidation to formate, followed by conversion to an amide intermediate (Scheme 1). In a next step, formamide decomposes to CO and NH_3_ or is dehydrated to HCN, the last reaction being more probable.^14^, ^19^ The observed formation of HCOOH (Figures 1 B, 2 and the Supporting Information) at low temperatures supports this alternative reaction path. Furthermore, it could be also linked to CO generation by dehydration, as observed for zeolite‐based catalysts.^20^ The impeding of the complete formaldehyde conversion to CO_2_ over Fe‐ZSM‐5, which could be formed by CO or HCOOH oxidation^21^ (Scheme 1), could be explained by the lack of redox active sites since the reoxidation of Fe^2+^ to Fe^3+^ is known to be a rate‐determining step during the SCR reaction,^22^ and in the present case is further inhibited by CO presence.

**Scheme 1:**
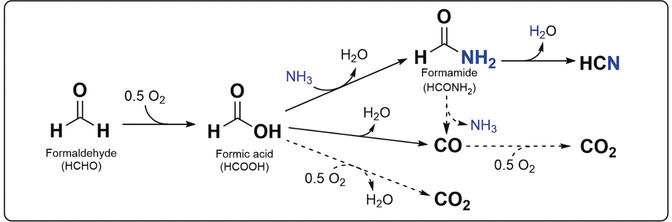
Suggested mechanism for HCN formation on state‐of‐the art catalysts for selective catalytic reduction of NO_*x*_ with NH_3_ under standard SCR conditions. Dotted arrows indicate less favored pathways.

This path involving the conversion of formic acid to formamide via reaction with NH_3_, as depicted in Scheme 1, is supported also by the DRIFTS measurements during HCHO and NH_3_ co‐adsorption on Fe‐ZSM‐5 at 150 °C (more details in the Supporting Information). The DRIFT spectrum of NH_3_ adsorbed on Fe‐ZSM‐5 show, for the spectral region reported here, the appearance of a main band around 1450 cm^−1^. This is in agreement with previous studies,^23^ indicating NH_3_ adsorption as NH_4_^+^ ions at the Brønsted acid sites. HCHO adsorption resulted in a dominant band around 1580 cm^−1^, previously attributed to the formation of formates at the Al or Fe sites of Fe‐ZSM‐5.^24^ The formation of formate on the Fe species is also supported by the studies of Viertelhaus et al.^25^ and of Johnson et al.^26^ on Fe^II^ and Fe^III^ formate complexes, with characteristic bands between 1586–1625 cm^−1^ due to asymmetric stretching frequencies of CO or OCO groups. The weaker bands appearing at 1321 cm^−1^, 1348 cm^−1^, 1369 cm^−1^ and 1402 cm^−1^ can be as well attributed to symmetric stretching in formates.^25^, ^27^ When dosing a combined gas mixture of NH_3_, HCHO and O_2_ on Fe‐ZSM‐5 additional bands were observed at 1666 cm^−1^, 1678 cm^−1^, 1691 cm^−1^, 1708 cm^−1^ and 1726 cm^−1^. With a minor or no shift, the most intense band at 1691 cm^−1^ was claimed by several studies as the fingerprint of adsorbed formamide.^28^ Further characteristic bands of formamide adsorption were also reported at lower or higher wavenumbers and were assigned to NH, NH_2_, CH or CO groups stretching on α‐Fe_2_O_3_,^29^ Fe_2_O_3_/SiO_2_ 28c and amorphous silica.28d These bands could be only partially identified in our study due to the overlap with other adsorbed species, particularly with formates. Hence, together with the detection of gaseous formic acid at low temperatures (Figure 2), the appearance of the bands characteristic for formates and formamide adsorption (Figure 3) clearly demonstrate the formation of these intermediate products of HCN emissions, supporting the mechanism suggested in Scheme 1.

**Figure 3:**
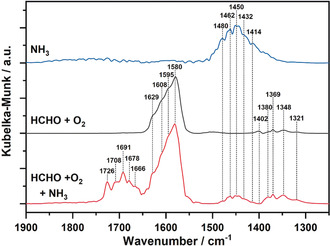
In situ DRIFTS spectra of Fe‐ZSM‐5 at 150 °C after exposure to NH_3_ (150 ppm NH_3_ in N_2_, blue line), HCHO + O_2_ (25 ppm HCHO, 5 % O_2_ in N_2_, black line) and HCHO + O_2_ + NH_3_ (25 ppm HCHO, 150 ppm NH_3_, 5 % O_2_ in N_2_, red line) and subsequent flushing in N_2_.

With small variations, the generation of HCN and CO secondary emissions during NH_3_‐SCR reaction in the presence of formaldehyde was uncovered also for all the other investigated catalysts. Table 1 reports the measured HCN and CO emissions (ppm values) at 250 °C and 500 °C for the four catalysts investigated in this study. For the same temperatures, the HCHO conversion and the HCN, CO and HCOOH yields are shown in Figure 4 (the difference to 100 % yield mainly corresponds to CO_2_). As in the case of Fe‐ZSM‐5, Fe‐BEA shows a similar share of selectivity for CO and HCN at low and high temperatures (Figures 4 and S11). In comparison with the iron zeolites, higher HCN emissions were produced on the V‐based sample over the whole temperature range, resulting in a maximum emission of 27 ppm at 500 °C (Figure 4 and Table 1). Only the Cu‐SSZ‐13 sample shows a significantly different emission profile and a pronounced drop of the low‐temperature SCR activity (Figures 4 and S16). Nonetheless, due to its superior low temperature performance (37 % formaldehyde oxidation at 250 °C in comparison to only 6 % conversion measured for Fe‐ZSM‐5) the absolute HCN and CO emission values are larger in this case, with a higher HCN share. This behavior is especially problematic since already at typical catalyst working temperatures around 250 °C significant amounts of HCN are formed. However, solely the Cu‐SSZ‐13 catalysts converts formaldehyde to CO and CO_2_ above 400 °C (about 70 % CO_2_ selectivity, Figures 4 and S16–S17), and no hydrogen cyanide could be detected.

**Figure 4:**
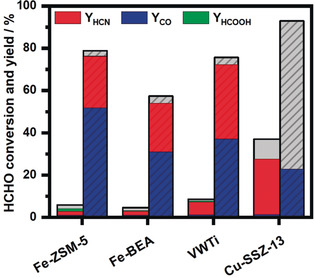
HCHO conversion and yield of toxic byproducts during standard SCR of NO_*x*_ with NH_3_ in presence of 80 ppm HCHO at 250 °C (plain columns) and 500 °C (cross‐striped columns). The difference in yield (grey area in the bar graph) mainly corresponds to CO_2_ formation.

**Table 1:** Formed emissions (ppm values) at two different temperatures in the presence of 80 ppm HCHO.

	Std SCR 250 °C		Fast SCR 250 °C		Std SC 500 °C		Fast SCR 500 °C	
	CO	HCN	CO	HCN	CO	HCN	CO	HCN
Fe‐ZSM‐5	1	2	1	10	40	19	46	14
Fe‐BEA	1	2	1	4	25	18	33	14
Cu‐SSZ‐13	1	19	1	30	17	0	21	0
VWTi	2	5	2	4	29	27	36	17

Since the exhaust gas aftertreatment system of a lean‐burn NG engine contains also an oxidation catalyst, NO oxidation to NO_2_ is an expected reaction.^4^ Hence, we also investigated the impact of NO_2_ presence on the secondary emission profile by testing the NH_3_‐SCR catalysts under fast SCR conditions (NO:NO_2_=1) for all four catalysts. For this gas mixture, slight formaldehyde and NH_3_ oxidation were measured above 500 °C as gas phase reactions (Figure S4). During the catalytic reaction, the influence of NO_2_ is significantly different depending on the catalyst formulation. Although the fast SCR reaction leads to a higher NO_*x*_ conversion in comparison to the standard SCR conditions, formaldehyde oxidation is not always positively affected. A comparison of CO and HCN emissions in ppm values under fast SCR conditions for 250 °C and 500 °C is shown in Table 1 for all four catalysts. An improvement of the HCHO oxidation activity was recorded for Fe‐ZSM‐5, Fe‐BEA and Cu‐SSZ‐13 (Figures S7, S11 and S17, about 30 % at 250 °C for Cu‐SSZ‐13) while a slight decrease of the low temperature performance was observed for the V‐based catalyst (Figure S14). Concurrently, in comparison to the standard SCR conditions higher HCN emissions were measured for both Fe‐exchanged zeolite catalysts and Cu‐SSZ‐13 at low temperatures but lower ones for the VWTi catalyst. At high temperatures, NO_2_ presence resulted in slightly decreased HCN concentrations and increased CO emissions for all samples. This difference is probably due to decomposition of NO_2_ to NO with generation of active oxygen radicals that oxidize formaldehyde in the gas phase, as demonstrated by the empty reactor test (Figure S4). NO_2_ could also help to faster reoxidize the Fe^2+^‐, Cu^+^‐ or V^4+^‐active centers,^30^ in this way promoting HCN conversion. Among the different catalyst formulations, the Cu‐SSZ‐13 sample seems to be the less problematic under both, standard and fast SCR conditions, since hydrogen cyanide is formed only in the low temperature range. Nonetheless, the high HCN emissions measured in this narrow temperature (Table 1) window are equally critical, considering the low catalytic efficiency of the proposed HCN removal catalysts at these temperatures.15b, 31

All in all, the poor activity of noble‐metal‐based catalysts to oxidize formaldehyde at low temperatures to CO_2_ under realistic reaction conditions8a, 8b, 9 (i.e., long‐term run and SO_2_ presence) and also the practically impossible complete conversion of formaldehyde even at high temperatures due to the too low diffusion rate,^10^ result in an inevitable exposure of the NO_*x*_‐removal catalysts to HCHO emissions. In this context, this study uncovers the formation of HCN as a potential major hazard during the application of conventional NH_3_‐SCR catalysts for NO_*x*_ removal in the exhaust of NG engines. Although such catalysts are commercially applied and considered highly efficient for reducing nitrogen oxides emissions, the presence of methane oxidation byproducts such as formaldehyde in the exhaust stream can lead to a very significant formation of the highly toxic hydrogen cyanide. In the worst case, we detected 30 ppm of HCN downstream of a Cu‐SSZ‐13 SCR catalyst at 200–250 °C under fast SCR conditions. In the high temperature regime and standard SCR conditions, about 27 ppm HCN were produced over a VWTi sample from 80 ppm HCHO dosed at the catalyst bed inlet. In order to remove HCN emissions, different materials have been proposed in literature,15b, 31, 32 some of them showing promising activity at high temperatures. However, on Pt‐based catalysts, which are typically present in the exhaust aftertreatment system to remove the potential NH_3_ slip emissions after the SCR catalyst, HCN is either converted with high selectivity to N_2_O and NO_*x*_ or is only poorly oxidized at low temperatures.15b, 31 Hence, without a feasible removal catalyst the high HCN yield as measured in this study under NG engine aftertreatment conditions represents a strong challenge for the state‐of‐the‐art NH_3_‐SCR catalysts and requires adequate measures to be taken. This is crucial especially when considering the increasing share of natural gas fueled cars, as predicted by the scenarios of the energy transition.

## Conflict of interest

The authors declare no conflict of interest.

## Supporting information

As a service to our authors and readers, this journal provides supporting information supplied by the authors. Such materials are peer reviewed and may be re‐organized for online delivery, but are not copy‐edited or typeset. Technical support issues arising from supporting information (other than missing files) should be addressed to the authors.

Supplementary
