# Supplementary material for: Emission of Toxic HCN During NOx Removal by Ammonia SCR in the Exhaust of Lean‐Burn Natural Gas Engines
Source: Angew Chem Int Ed Engl. 2020 Jul 1;59(34):14423–8. doi: 10.1002/anie.202003670 (PMC7497226; doi:10.1002/anie.202003670)
Supplement: Supplementary file 1 — Supplementary [file ANIE-59-14423-s001.pdf]

Angewandte Chemie
Eine Zeitschrift der Gesellschaft Deutscher Chemiker

Supporting Information

Emission of Toxic HCN During NO_x Removal by Ammonia SCR in the Exhaust of Lean-Burn Natural Gas Engines

Deniz Zengel, Pirmin Koch, Bentolhoda Torkashvand, Jan-Dierk Grunwaldt, Maria Casapu, and Olaf Deutschmann**

anie_202003670_sm_miscellaneous_information.pdf

Table of Contents

Experimental Procedures 1
Catalyst preparation 1
Catalyst characterization 1
In-situ Diffuse Reflectance Infrared Fourier Transform Spectroscopy (DRIFTS) 2
Test bench and procedure 2
Results and Discussion 2
Material Characterization 2
Gas Phase Reaction Data 3
Catalytic Activity Data 4
Fe-ZSM-5 5
Fe-BEA 6
V_2O_5-WO_3/TiO_2 (VWTi) 8
Cu-SSZ-13 9
References 11
Author Contributions 11

Experimental Procedures

Catalyst preparation

Four different types of SCR catalysts were prepared and coated on cordierite honeycombs: Cu-SSZ-13, Fe-BEA, Fe-ZSM-5 and V_2O_5-WO_3/TiO_2. The Cu-SSZ-13 was prepared by ion exchange of NH_4-SSZ-13 (Si/Al = 15) zeolite with a 0.005 molar aqueous solution of $Cu(CH_3COO)_2$ at room temperature for 24 hours, as described in the study of Günter et al.[1] Subsequently, the sample was washed with deionized water and dried at 70 °C in static air. The resulting sample was calcined in air at 550°C for 4 hours. In the case of Fe-exchanged zeolites, H-BEA (Si/Al = 13) and NH_4-ZSM-5 (Si/Al = 12), the ion exchange was performed with a 0.0024 molar aqueous solution of $Fe(NO_3)_3 \cdot 9H_2O$ at 80 °C for 48 hours, similar to the study of Lablonska et al.[2] The obtained samples were washed, dried and calcined as described above. For the V-based catalyst, the incipient wetness impregnation method was applied to add tungsten ($(NH_4)_6H_2W_{12}O_{40} \cdot xH_2O$) and vanadium ($NH_4VO_3$) precursors to TiO_2, based on the work of Japke et al.[3] After impregnation the catalyst was dried at 70 °C and calcined in air at 550 °C for 4 hours.
In a next step, the resulting powders were added to cordierite honeycombs (cell density 400 cpsi, 2.54 cm in diameter and 3 cm in length) via dip coating. The catalyst slurries were prepared by mixing the catalyst powders either with 8 wt% of Disperal P2 (Sasol) for Cu- and Fe-exchanged zeolites or with 8 wt% LUDOX AS-40 for V-catalyst, amount added relative to the total mass to enhance the adhesion, and 45 mL of demineralized water. After several coating and drying steps, the desired loading of the honeycomb was achieved. The coated honeycomb was calcined in static air at 550 °C for 4 hours. The amount of added catalytic active washcoat is listed in Table S1.

Table S1. Amount of applied washcoat for each honeycomb.

	V_2O_5-WO_3/TiO_2	Fe-ZSM-5	Fe-BEA	Cu-SSZ-13
**Washcoat mass [g]**	2.42	2.44	2.38	2.58

Catalyst characterization

XRD patters were collected for the as prepared catalyst powders using a D8 Advanced X-ray diffractometer from Bruker with Cu Kα radiation. The scans were recorded over a 2θ range of 20-80° with steps of 0.017° for the V-catalyst or a 2θ range of 8-50° with steps of 0.033° for zeolites. A Belsorp Mini II instrument (Bel Japan Inc.) was used to determine the surface area and the pore volume of the catalysts. Prior to the analysis, approximately 80 mg of the sample were degassed at 300 °C under vacuum for 2 hours. Afterwards, adsorption and desorption of N_2 was measured and evaluated with the Belsorp Data Analysis Software using the Brunauer-Emmet-

Teller isotherm.[4] The elemental composition was identified by X-ray fluorescence (XRF) analysis at the Institute for Applied Materials (IAM, KIT).

In-situ Diffuse Reflectance Infrared Fourier Transform Spectroscopy (DRIFTS)

The DRIFTS measurements were conducted at 150 °C for Fe-ZSM-5 with a VERTEX 70 FTIR spectrometer (Bruker) equipped with Praying Mantis diffuse reflection optics (Harrick), a liquid nitrogen-cooled mercury cadmium telluride detector and a high-temperature *in-situ* cell (Harrick). The sample was diluted with CaF_2 to gain a mixture of 5/95 catalyst to CaF_2. After diluting, the sample was pressed and sieved to a grain size of 100-200 µm. The sample, approximately 50 mg, was placed in a cup and the high temperature *in-situ* cell was closed with a CaF_2 window. The resulting gaseous mixture at the reactor outlet was monitored with a mass spectrometer (ThermoStar, Pfeiffer Vacuum). Since the *in-situ* DRIFTS experiments were to be carried out in the absence of water, a gas bottle (50 ppm HCHO in N_2, Guttroff) was used in this case for formaldehyde dosing. Before the experiment, the catalyst was baked out in 5% O_2 in N_2 at 400 °C for 1 hour and subsequently cooled down to 150 °C in N_2. The background spectrum was taken at 150 °C in N_2. Afterwards gas mixtures consisting of 25 ppm HCHO, 5% O_2 in N_2 (HCHO + O_2), 25 ppm HCHO, 150 ppm NH_3, 5% O_2 in N_2 (HCHO + O_2 + NH_3) or 150 ppm NH_3 in N_2 were used to saturate the surface of the catalyst for 30 minutes. After each exposure the cell was flushed with N_2 for 1 hour, followed by spectra acquisition. As reference for adsorbed NH_3 a fresh sample was used and pretreated as mentioned above. DRIFTS spectra were recorded in reflectance mode and converted with the Kubelka-Munk function.

Test bench and procedure

The catalytic activity tests were performed on a test bench of the Exhaust Center Karlsruhe. The test bench consists of gas dosage mass flow controllers, a counter-flow steel reactor and an MKS Multigas 2030 FTIR spectrometer for gas analytics. Water was added to the main gas stream with a controlled evaporator mixer (CEM). In order to dose formaldehyde, an aqueous solution of paraformaldehyde (16% solution, EM grade, Electron Microscopy Science) was used, which was dosed with a heated gas saturator. All gas tubes were heated to a temperature of 175 °C to prevent condensation before and after the catalyst.
Before each series of experiments, the fresh catalysts were conditioned for 2 h at 550 °C in standard SCR gas mixture (350 ppm NO, 350 ppm NH_3, 12% H_2O, 10% O_2 and N_2 balance). The conditioning as well as the following activity tests were conducted under a total flow of about 25 L/min that corresponds to a gas hourly space velocity (GHSV) of 100,000 h^{-1}. The catalysts were tested for their NO oxidation (350 ppm NO, 12% H_2O, 10% O_2 and N_2 balance), NH_3 oxidation (350 ppm NH_3, 12% H_2O, 10% O_2 and N_2 balance), standard SCR (350 ppm NO, 350 ppm NH_3, 12% H_2O, 10% O_2 and N_2 balance) and fast SCR (175 ppm NO, 175 ppm NO_2, 350 ppm NH_3, 12% H_2O, 10% O_2 and N_2 balance) steady-state activities in a stepwise manner between 550 °C and 150 °C, with 50 °C steps. All gas mixtures were tested without HCHO first and afterwards in presence of 80 ppm HCHO. In addition, also the HCHO oxidation with 12% H_2O and 10% O_2 in N_2 was measured. To exclude any involvement of gas phase reactions at higher temperatures, empty reactor tests were performed using the above-mentioned reaction conditions in absence of a catalyst.
The conversion X of the different compounds was calculated with the following formula:
In this equation c_{in} stands for the inlet concentration, which was measured on bypass of the reactor. The concentration c_{out} was measured after the reactor with a FTIR gas analyzer.

$$X = \frac{c_{in} - c_{out}}{c_{in}} * 100$$

Since all the resulting products of HCHO conversion consisted of the same amount of carbon, which means 1 ppm HCHO would form 1 ppm CO/HCN/HCOOH, the selectivity S for was calculated with following equation (for CO as an example):

$$S_{CO,HCHO} = \frac{c_{CO,in}}{c_{HCHO,in} - c_{HCHO,out}} * 100$$

Results and Discussion

Material Characterization

All results of N_2 physisorption, elemental analysis and x-ray diffraction are shown in figures S1 and S2 as well as in table S2. In case of the impregnated V_2O_5-WO_3/TiO_2 catalyst a loading of 1.1 wt% V and 7.0 wt% W was detected, which corresponds to a load of 2.0 wt% V_2O_5 and 8.8 wt% WO_3.

Table S2. Summary of catalyst properties determined by elemental analysis (XRF) and N_2 physisorption (BET).

	V_2O_5-WO_3/TiO_2			Fe-ZSM-5			Fe-BEA			Cu-SSZ-13		
**Elemental composition [wt%]**	V	W	Ti	Fe	Si	Al	Fe	Si	Al	Cu	Si	Al
	1.10	7.04	51.4	1.27	39.9	3.10	1.35	39.8	2.85	1.67	39.9	2.60
**Surface area [m²/g]**		80			410			600			810	
**Pore volume [mL/g]**		0.2			0.2			0.6			0.3	

In case of the zeolite-based catalysts only the characteristic XRD patterns of SSZ-13, BEA or ZSM-5 supports were obtained (Figure S1), indicating the absence of large copper and iron oxide particles. Also, for the impregnated V_2O_5-WO_3-TiO_2 catalyst, only TiO_2 anatase phase could be identified (Figure S2), which suggest a good distribution of WO_3 and V_2O_5 species.

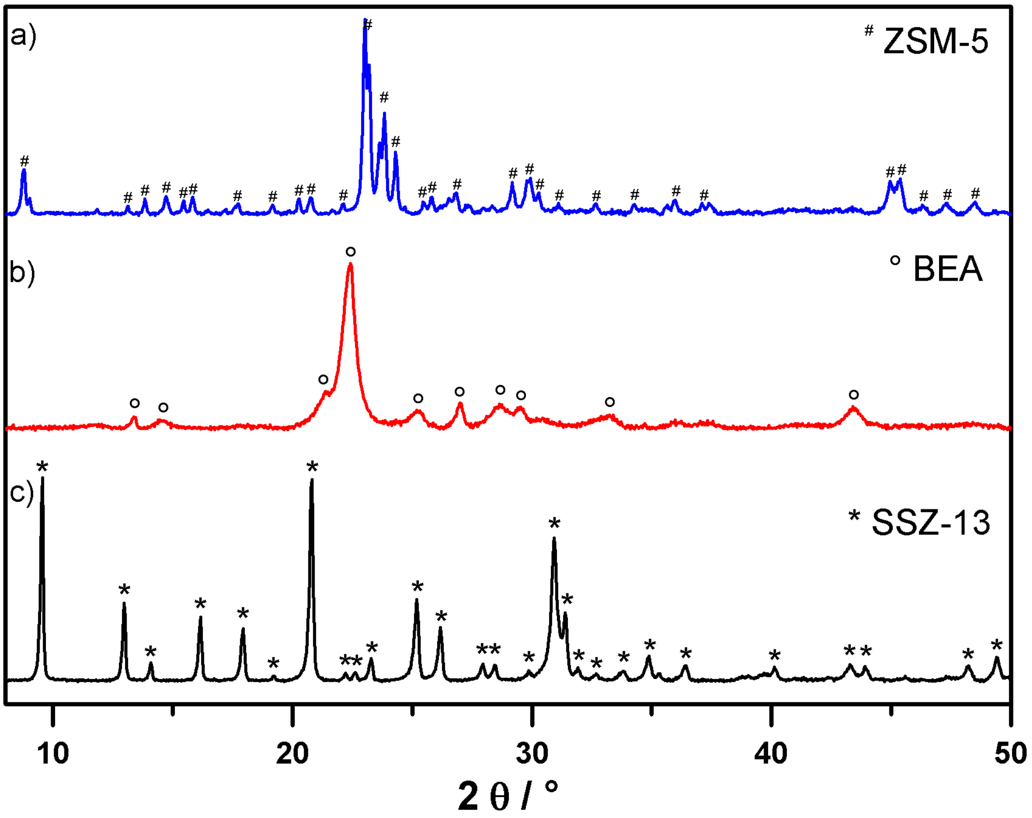

Figure S1. XRD patterns of a) Fe-ZSM-5, b) Fe-BEA and c) Cu-SSZ-13 after ion exchange.[1,5,6]

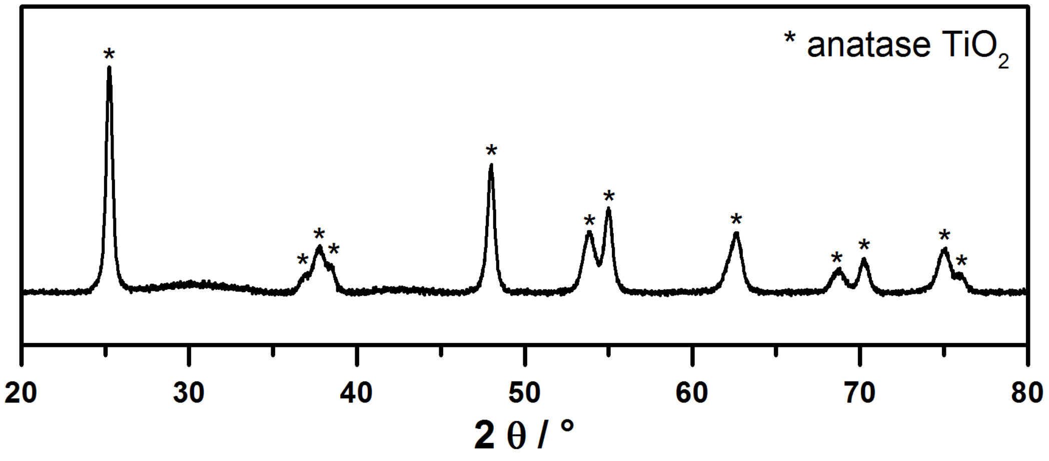

Figure S2. XRD pattern of the impregnated V_2O_5-WO_3/TiO_2.[7]

Gas Phase Reaction Data

In order to exclude influences of gas phase reactions, empty reactor measurements (absence of catalyst) with standard SCR (Figure S3) and fast SCR (Figure S4) gas mixtures were carried out before the catalytic measurements. During standard SCR (Figure S3) the gas composition was stable up to a temperature of 550 °C. Only small amounts of NH_3 and HCHO were converted at 550 °C and a slight increase in NO emission was observable. There was also no formation of HCN or CO, which could interfere with the emissions formed over the catalyst.

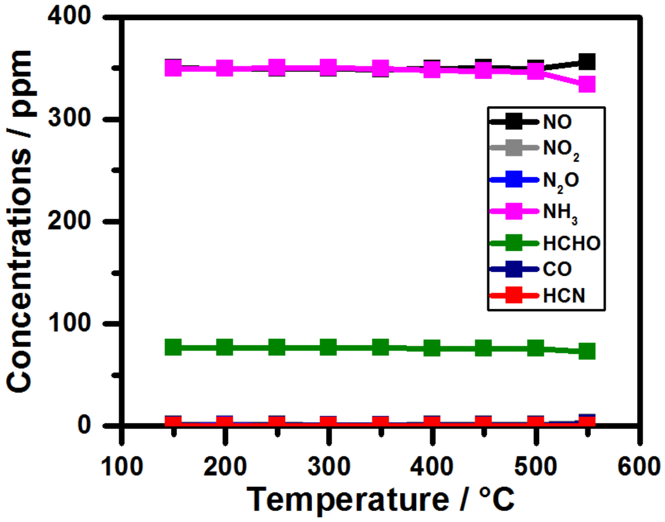

Figure S3. Empty reactor test with standard SCR gas mixture consisting of 350 ppm NO, 350 ppm NH_3, 80 ppm HCHO, 12% H_2O and 10% O_2 in N_2 balance and a total gas flow of 25.3 L/min.

In the presence of NO_2 (fast SCR, Figure S4) a slight change of gas composition above 500 °C was observed. Compared to standard SCR gas mixture, the conversion of NH_3 and HCHO was slightly enhanced. Also, small amounts of CO were formed at the highest temperature (550 °C). Since for both gas mixtures only minor influences of gas phase reactions were observed and no traces of HCN could be detected, the impact of those reactions were considered negligible.

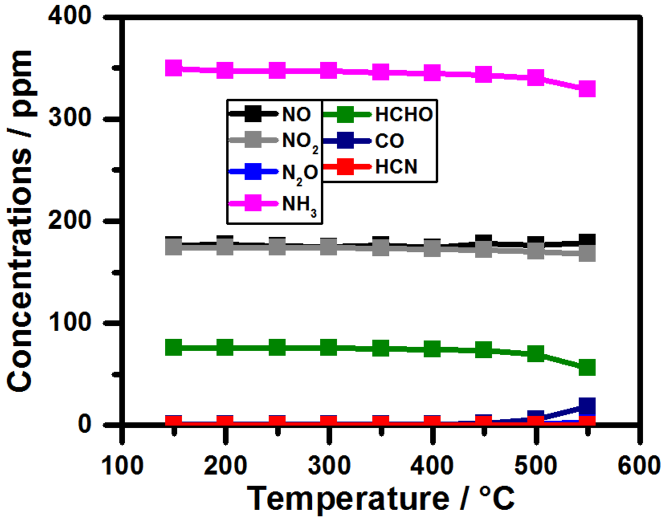

Figure S4. Empty reactor test with fast SCR gas mixture consisting of 175 ppm NO, 175 ppm NO_2, 350 ppm NH_3, 80 ppm HCHO, 12% H_2O and 10% O_2 in N_2 balance and a total gas flow of 25.3 L/min.

Catalytic Activity Data

Fe-ZSM-5

For all four catalysts, measurements in the presence and absence of HCHO (HCHO oxidation, standard SCR and fast SCR) were conducted. In Figure S6 the HCHO oxidation over Fe-ZSM-5 in a mixture consisting of 12% H_2O, 10% O_2 and N_2 is shown. Fe-ZSM-5 was able to oxidize HCHO above 350 °C and reached a maximal conversion of 81% at 550 °C. The main product of HCHO oxidation was CO. Even at the highest temperature (550 °C) all converted HCHO was oxidized to CO.

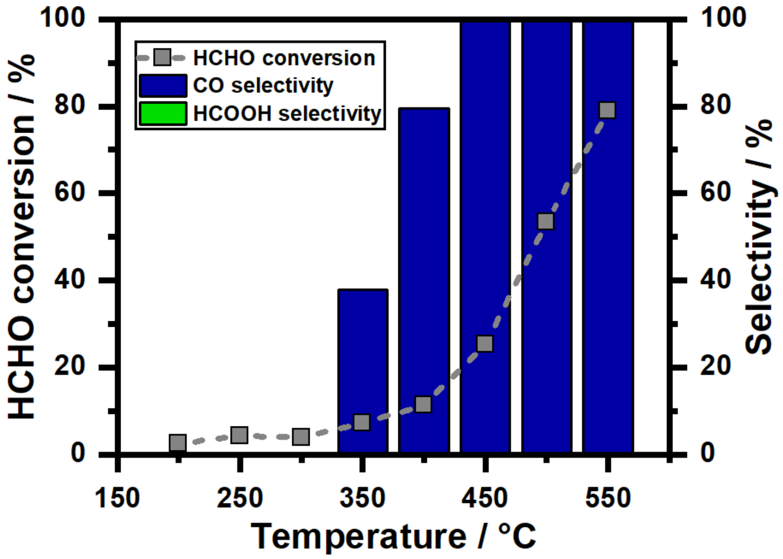

Figure S5. HCHO Oxidation and product selectivity over Fe-ZSM-5 with a gas mixture consisting of 80 ppm HCHO, 12% H_2O, 10% O_2 in N_2 balance and a gas hourly space velocity of 100,000 h^{-1}.

In the presence of NO and NH_3 (standard SCR, Figures 1 and S6) the HCHO conversion is enhanced. A negative impact on NO_x conversion was visible only above 400 °C. With increasing temperature, the negative influence of formaldehyde is even more pronounced up to an activity loss of 12% at 550 °C. Although NO_x conversion decreased, an additional consumption of NH_3 was observed, due to the formation of HCN. For the Fe-ZSM-5 catalyst, HCN was formed over the whole temperature range with a selectivity up to 50%. At low temperatures small amounts of HCOOH were detected.

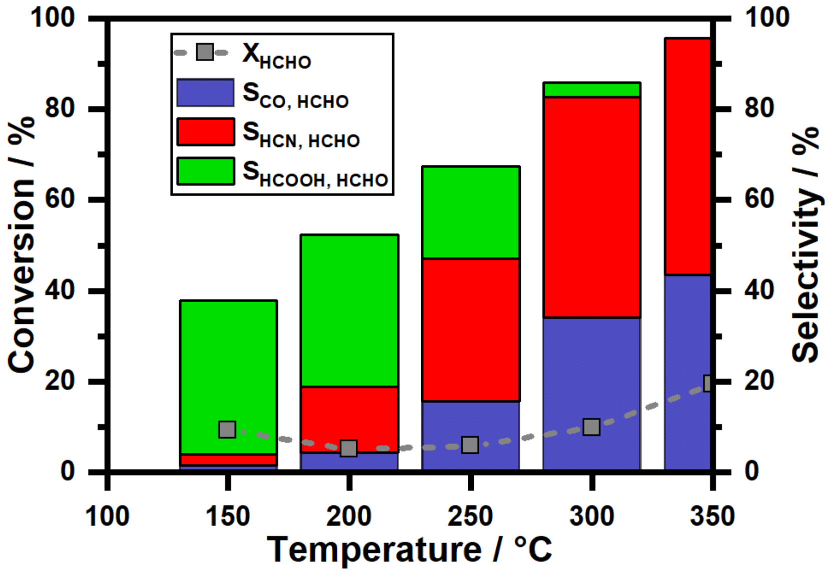

Figure S6. HCHO conversion and product selectivity of Fe-ZSM-5 during standard SCR from 150 °C to 350 °C in a gas mixture consisting of 350 ppm NO, 350 ppm NH_3, 80 ppm HCHO, 12% H_2O, 10% O_2 in N_2 balance and a gas hourly space velocity of 100,000 h^{-1}.

SUPPORTING INFORMATION

The conversion and selectivity during fast SCR conditions for Fe-ZSM-5 are depicted in Figure S7. Formaldehyde seemed to influence the NO_x conversion activity over the whole temperature range. The NO_x conversion as well as the NH_3 oxidation decreased approximately by 8%. The presence of NO_2 seemed to favor the formation of HCN especially at lower temperatures. In addition, fast SCR conditions enhanced the HCHO conversion over the whole temperature range.

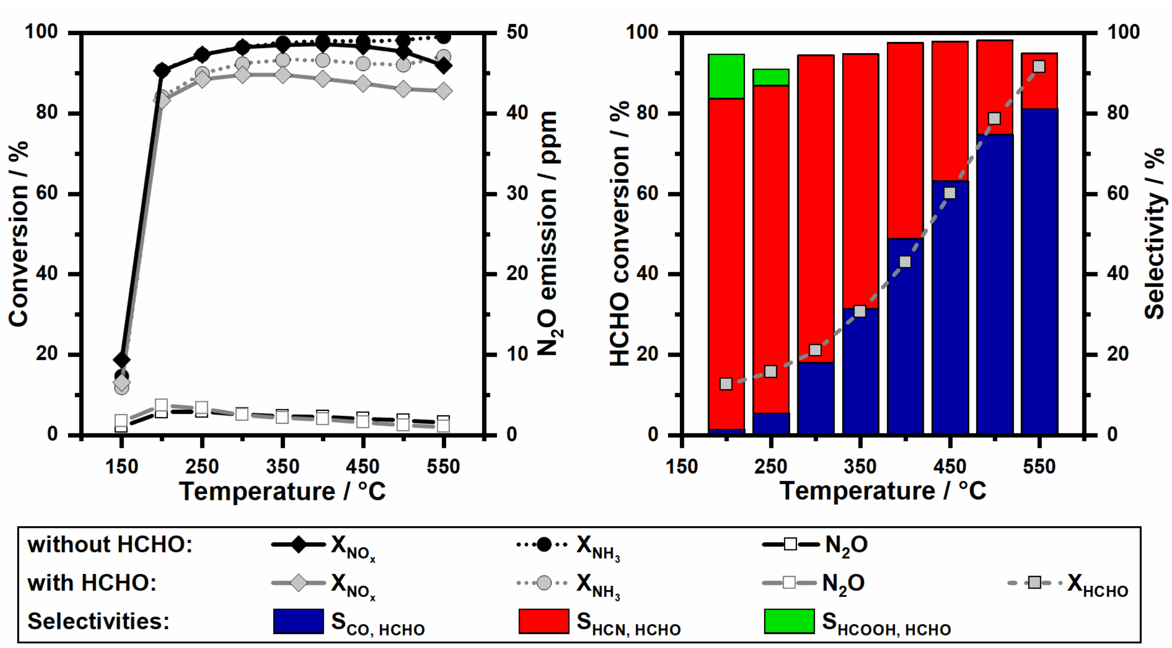

Figure S7. Comparison of NO_x and NH_3 conversion during fast SCR over Fe-ZSM-5 with and without formaldehyde in a gas mixture consisting of 175 ppm NO, 175 ppm NO_2, 350 ppm NH_3, 0-80 ppm HCHO, 12% H_2O, 10% O_2 in N_2 balance and a gas hourly space velocity of 100,000 h^{-1}(left). HCHO conversion and product selectivity during fast SCR conditions (right).

To exclude any involvement of CO in the formation of HCN and also to test the ability of Fe-ZSM-5 to oxidize CO to CO_2 a measurement under standard SCR conditions in the presence of 100 ppm CO was conducted (Figure S8). During this test no emissions of HCN were observed. Furthermore, only small amounts of CO were oxidized to CO_2 at temperatures above 350 °C, confirming that Fe-ZSM-5 was a rather poor oxidizing catalyst.

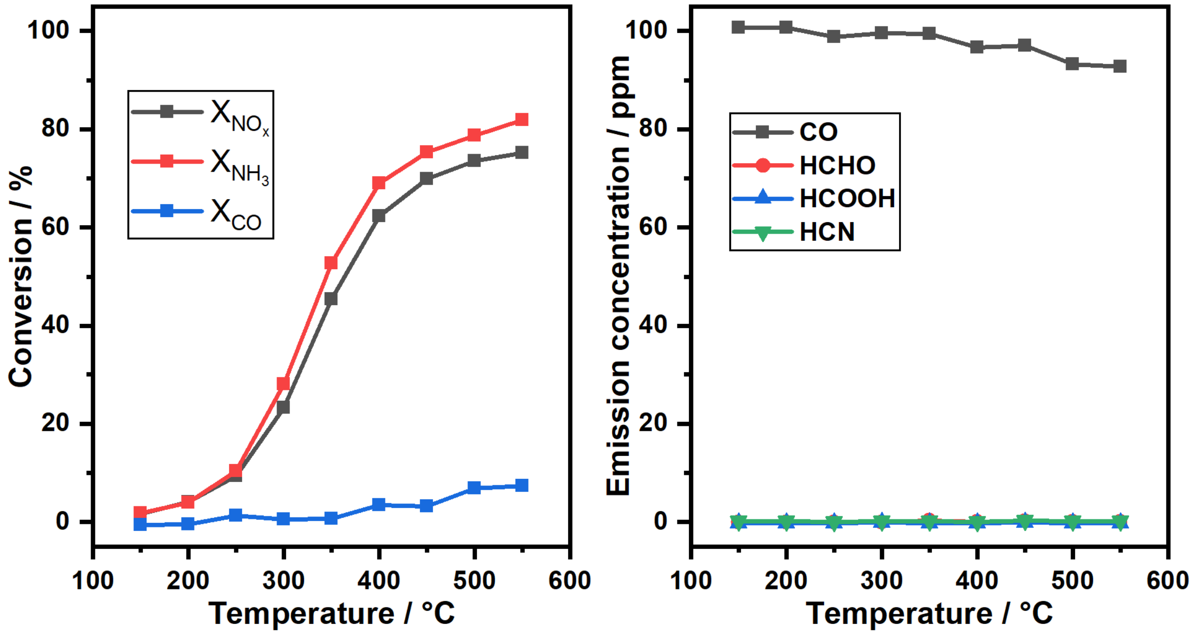

Figure S8. NO_x, NH_3 and CO conversion during standard SCR over Fe-ZSM-5 in a gas mixture consisting of 350 ppm NO, 350 ppm NH_3, 100 ppm CO, 12% H_2O, 10% O_2 in N_2 balance and a gas hourly space velocity of 100,000 h^{-1}(left). Emission of carbonaceous species during CO conversion in standard SCR gas mixture (right).

Fe-BEA

In the case of Fe-BEA, a lower HCHO oxidation activity was recorded compared to Fe-ZSM-5, and the conversion of formaldehyde started above 350 °C (**Fehler! Verweisquelle konnte nicht gefunden werden.** S9). Even at the highest temperature the catalyst was only able to convert half of the HCHO. As in the case of Fe-ZSM-5, the majority of HCHO was converted into CO, particularly above 400 °C.

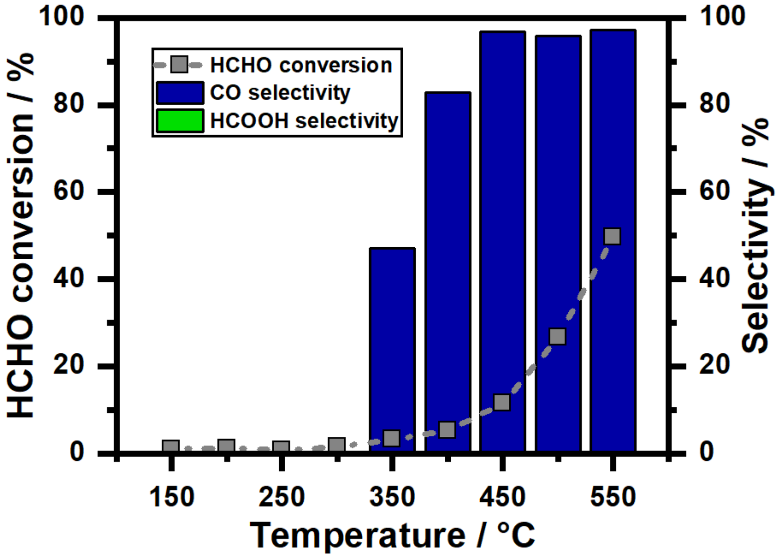

Figure S9. HCHO Oxidation and product selectivity over Fe-BEA with a gas mixture consisting of 80 ppm HCHO, 12% H_2O, 10% O_2 in N_2 balance and a gas hourly space velocity of 100,000 h^{-1}.

The NO_x conversion during standard SCR (Figure S10) over Fe-BEA showed a minor impact of formaldehyde presence. Only a slightly increased NH_3 consumption was visible over the whole temperature range and a decreased NO_x conversion above 400 °C. Above 350 °C the selectivity towards CO formation is constantly higher than 50%. For this sample HCN emissions were recorded even at the highest investigated temperature.

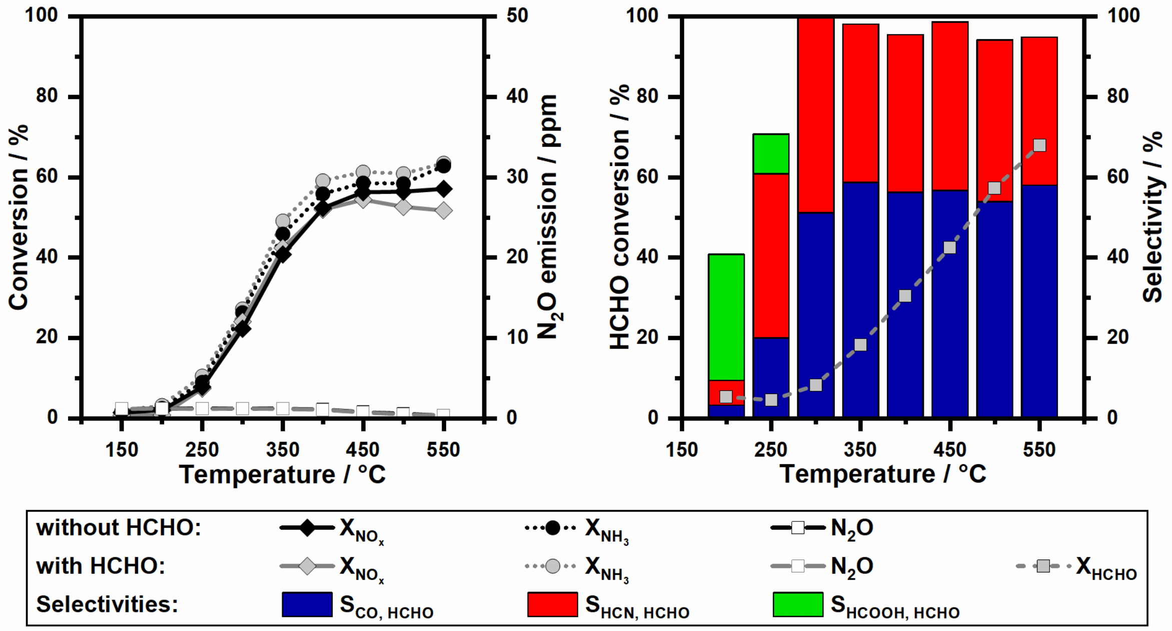

Figure S10. Comparison of NO_x and NH_3 conversion during standard SCR over Fe-BEA with and without HCHO in a gas mixture consisting of 350 ppm NO, 350 ppm NH_3, 0-80 ppm HCHO, 12% H_2O, 10% O_2 in N_2 balance and a gas hourly space velocity of 100,000 h^{-1}(left). HCHO conversion and product selectivity during standard SCR conditions (right).

SUPPORTING INFORMATION

In the presence of NO_2 (fast SCR, Figure S11) the oxidation of HCHO was enhanced at high temperatures. Increasing temperature shifted the selectivity of HCHO oxidation products from HCN towards CO up to 70% share of CO at the highest temperature. NO_x conversion slightly decreased at all investigated temperatures, and especially at 200°C and above 350 °C. Similar as under standard SCR conditions, HCN was formed over the whole temperature range leading to a higher NH_3 conversion compared to NO_x conversion.

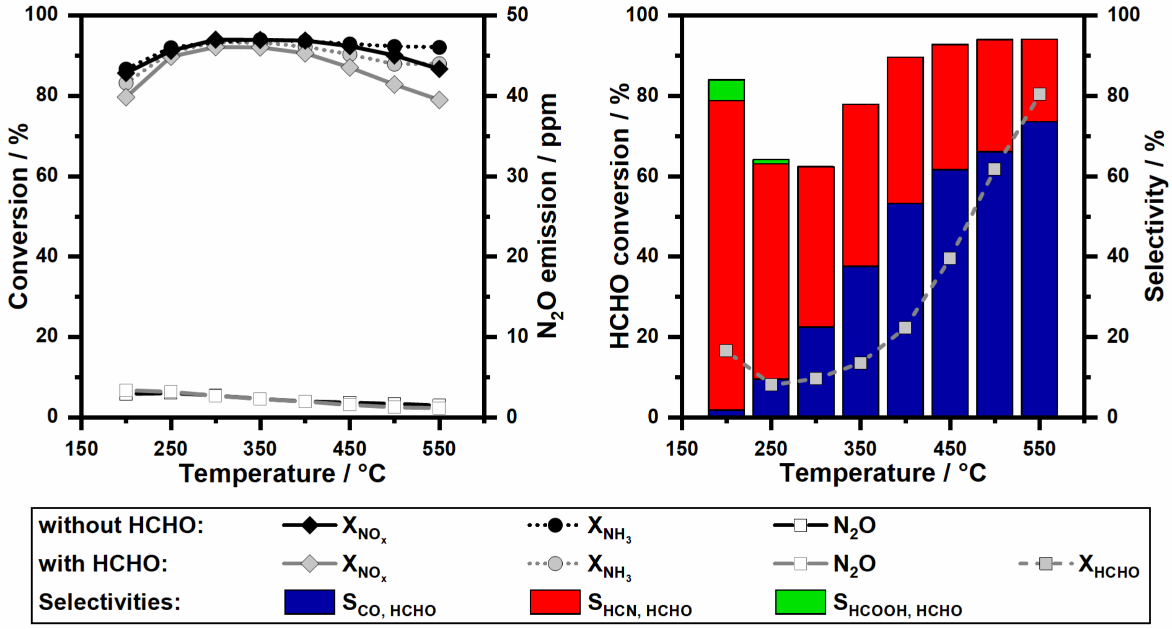

Figure S11. Comparison of NO_x and NH_3 conversion during fast SCR over Fe-BEA with and without formaldehyde in a gas mixture consisting of 175 ppm NO, 175 ppm NO_2, 350 ppm NH_3, 0-80 ppm HCHO, 12% H_2O, 10% O_2 in N_2 balance and a gas hourly space velocity of 100,000 h^{-1}(left). HCHO conversion and product selectivity during fast SCR conditions (right).

V_2O_5-WO_3/TiO_2 (VWTi)

Another tested common SCR catalyst was the V_2O_5-WO_3/TiO_2 (VWTi). During HCHO oxidation (Figure S12), this catalyst showed a rather poor catalytic activity. The maximum conversion was 70% at 550 °C. Above 250°C almost all converted HCHO formed CO. Only at low temperatures low formic acid emissions were observed.

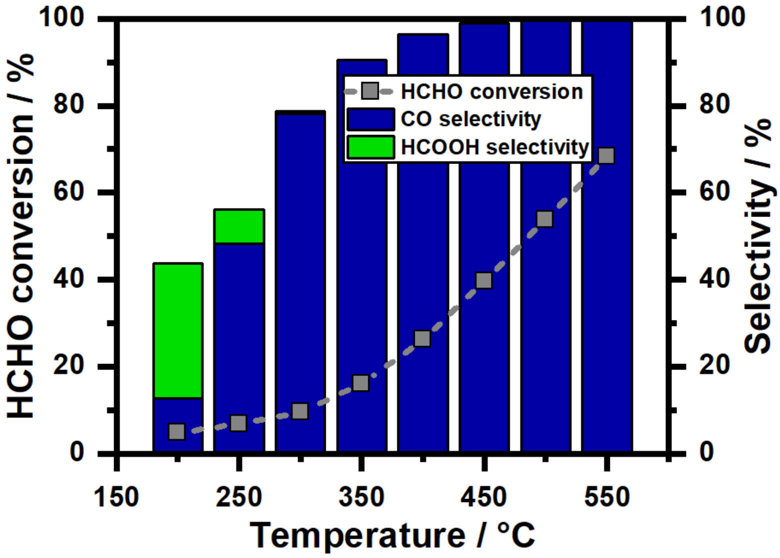

Figure S12. HCHO Oxidation and product selectivity over VWTi with a gas mixture consisting of 80 ppm HCHO, 12% H_2O, 10% O_2 in N_2 balance and a gas hourly space velocity of 100,000 h^{-1}.

Under standard SCR conditions HCHO (Figure S13) oxidation was enhanced over the whole temperature range. The majority of the products consisted of HCN and CO but the share was shifted towards CO formation. It is also notable that the conversion of formaldehyde was lower as that measured in the absence of NO. On the contrary, the conversion of NO_x is slightly decreased (about 5% at 350°C) under these conditions. In the case of N_2O emissions, no influence of HCHO presence was visible.

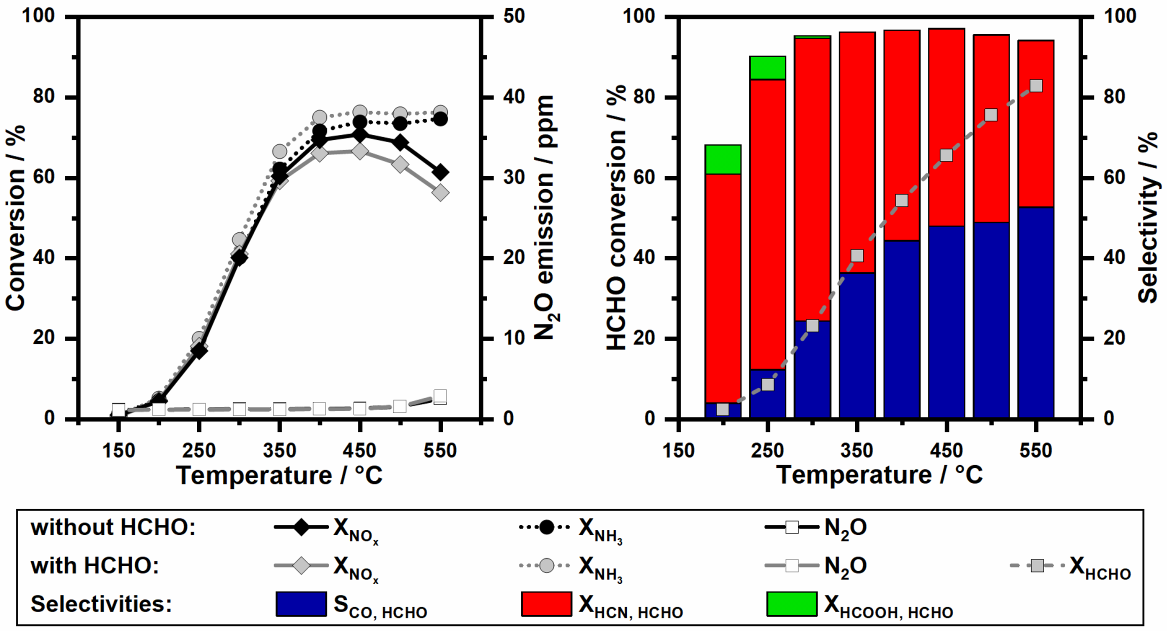

Figure S13. Comparison of NO_x and NH_3 conversion during standard SCR over VWTi with and without HCHO in a gas mixture consisting of 350 ppm NO, 350 ppm NH_3, 0-80 ppm HCHO, 12% H_2O, 10% O_2 in N_2 balance and a gas hourly space velocity of 100,000 h^{-1}(left). HCHO conversion and product selectivity during standard SCR conditions (right).

Under fast SCR conditions (Figure S14) an increase of NO_x conversion in the low temperature region was measured. Above 300 °C the catalytic activity was diminished in the presence of formaldehyde. N_2O emissions were the same with and without HCHO, except for the highest investigated temperature where a slight increase was observed. The HCHO oxidation was slightly reduced under these conditions and the selectivity was shifted more towards CO compared to that recorded for the standard SCR gas mixture.

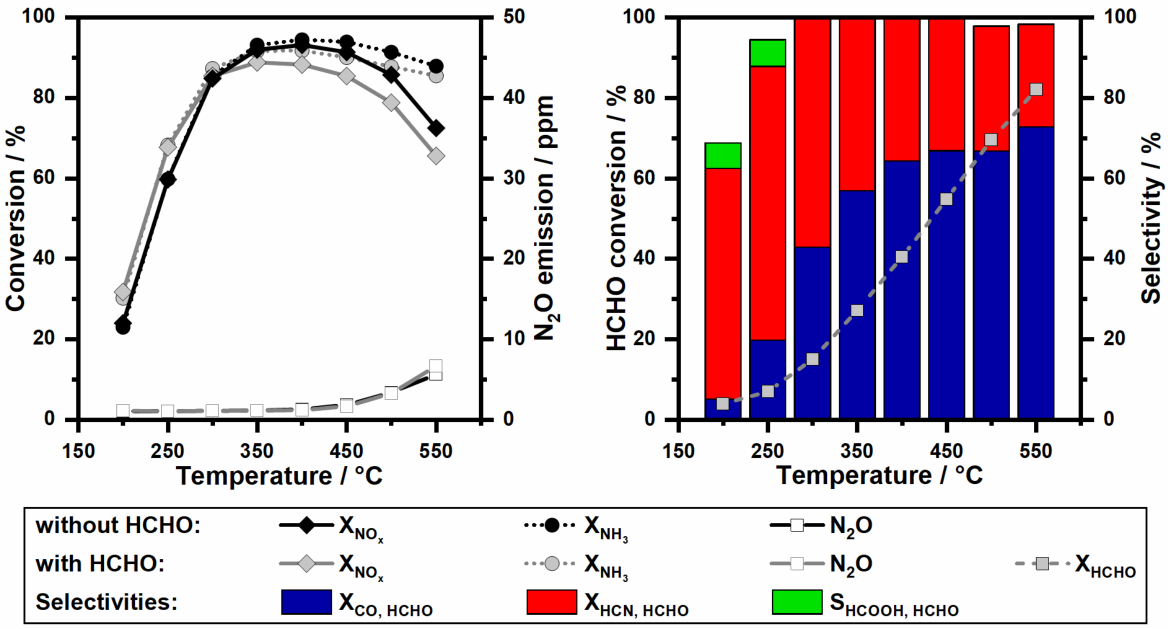

Figure S14. Comparison of NO_x and NH_3 conversion during fast SCR over VWTi with and without formaldehyde in a gas mixture consisting of 175 ppm NO, 175 ppm NO_2, 350 ppm NH_3, 0-80 ppm HCHO, 12% H_2O, 10% O_2 in N_2 balance and a gas hourly space velocity of 100,000 h^{-1}(left). HCHO conversion and product selectivity fast SCR conditions (right).

Cu-SSZ-13

The fourth tested catalyst Cu-SSZ-13 showed a significantly different behavior compared to the other samples. In Figure S15 the HCHO conversion in a mixture consisting of 80 ppm HCHO, 12% H_2O, 10% O_2 and N_2 is shown. The conversion of HCHO started at around 250 °C and reaches 50% HCHO conversion between 350 and 400 °C. At 550 °C almost full conversion of HCHO is achieved. The selectivity to CO was rather low compared to the other catalysts and increased with rising temperature up to a maximum of 35% at 500°C. Since no other byproducts were detected we assume that the rest of the conversion products consists of CO_2.

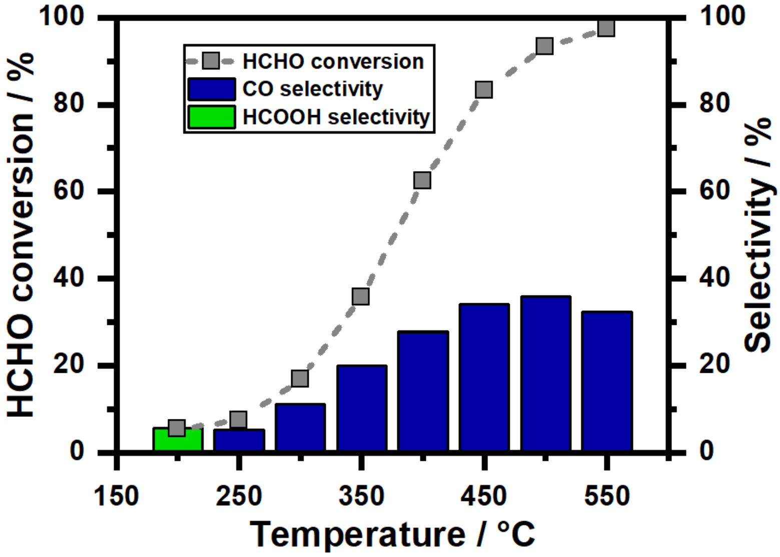

Figure S15. HCHO Oxidation and product selectivity over Cu-SSZ-13 with a gas mixture consisting of 80 ppm HCHO, 12% H_2O, 10% O_2 in N_2 balance and a gas hourly space velocity of 100,000 h^{-1}.

The influence of formaldehyde on the standard SCR reaction over Cu-SSZ-13 is shown in Figure S16. An increased conversion of HCHO was observed at low and intermediate temperatures (150-400 °C). In this temperature regime the selectivity to HCN reached a selectivity up to 63%. Beside the formation of large amounts of toxic HCN, the HCHO also impacted the conversion of nitrogen oxides. In presence of HCHO NO_x conversion was reduced up to a maximum decrease of 41% at 250 °C, suggesting HCN formation as the favored reaction over Cu active sites. Simultaneously to the loss of NO_x conversion also N_2O emission declined in the low temperature region. At temperatures above 400 °C on which no HCN was produced anymore, the NO_x and NH_3 conversion was identical to the reference measurement without HCHO.

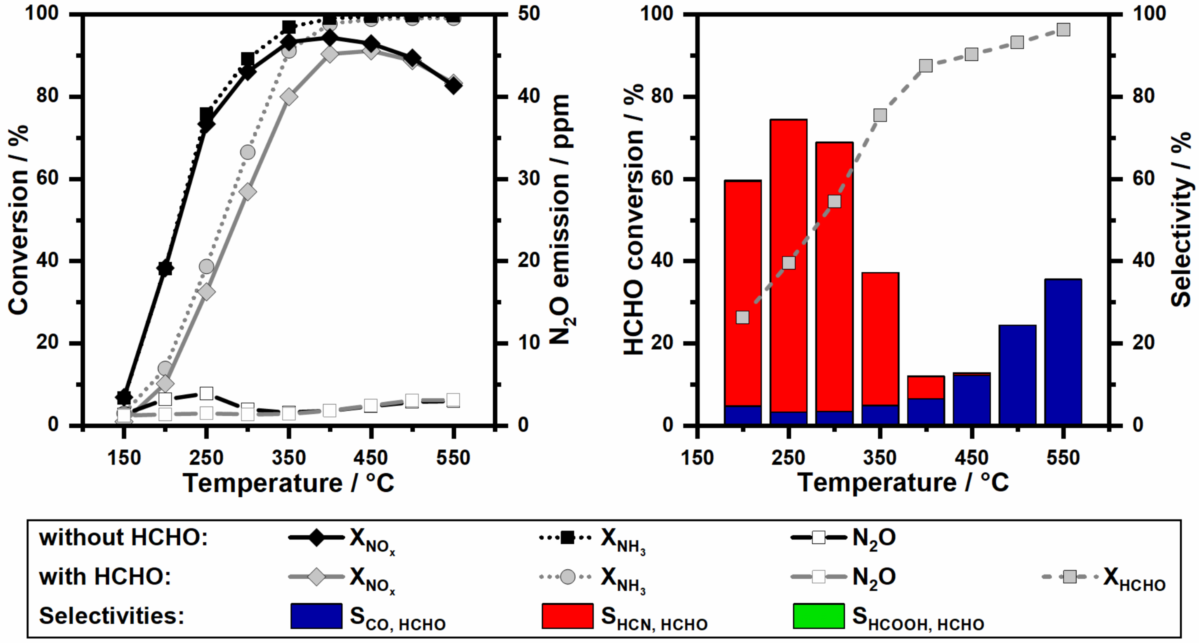

Figure S16. Comparison of NO_x and NH_3 conversion during standard SCR over Cu-SSZ-13 with and without HCHO in a gas mixture consisting of 350 ppm NO, 350 ppm NH_3, 0-80 ppm HCHO, 12% H_2O, 10% O_2 in N_2 balance and a gas hourly space velocity of 100,000 h^{-1}(left). HCHO conversion and product selectivity during standard SCR conditions (right).

In presence of NO_2 (Figure S17), the formaldehyde conversion was significantly increased with more than 90% at 350 °C. The NO_x conversion and N_2O emissions during fast-SCR decreased at temperatures below 450 °C, where the HCN formation took place. In general, the selectivity to CO and HCN showed the same trend as for the standard SCR: the formation of HCN at temperatures below 250 °C was enhanced. Although the NO_x conversion decrease was not as pronounced as during standard SCR, the formation of HCN seemed to be the favored reaction over the Cu active sites in the low temperature regime. Although Cu-SSZ-13 has shown the best NO_x removal activity and the most complete oxidation of HCHO, the large HCN selectivity at low temperature is a problem especially considering the low conversion of HCHO at those temperatures.

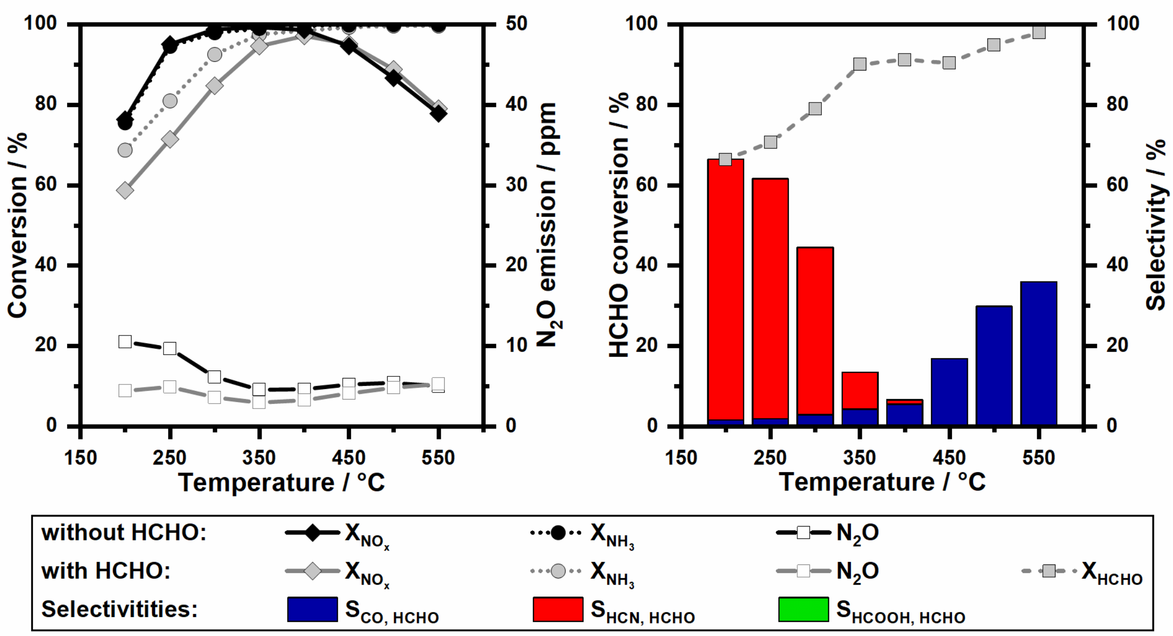

Figure S17. Comparison of NO_x and NH_3 conversion during fast SCR over Cu-SSZ-13 with and without formaldehyde in a gas mixture consisting of 175 ppm NO, 175 ppm NO_2, 350 ppm NH_3, 0-80 ppm HCHO, 12% H_2O, 10% O_2 in N_2 balance and a gas hourly space velocity of 100,000 h^{-1}(left). HCHO conversion and product selectivity during fast SCR conditions (right).

References

[1] T. Günter, H. W. Carvalho, D. E. Doronkin, T. Sheppard, P. Glatzel, A. J. Atkins, J. Rudolph, C. R. Jacob, M. Casapu, J. -D. Grunwaldt, *Chem. Commun*. **2015**, *51*, 9227-9230.
[2] G. D. M. Jablonska, K. Kruczala, A. Blachowski, K. A. Tarach, K. Brylewska, C. Petitto, K. Gora-Marek, *J. Phys. Chem. C* **2016**, *120*, 16831-16842.
[3] E. Japke, M. Casapu, V. Trouillet, O. Deutschmann, J. -D. Grunwaldt, *Catal. Today* **2015**, *258*, 461-469.
[4] S. Brunauer, P. H. Emmett, E. Teller, *J. Am. Chem. Soc.* **1938**, *60*, 309-319.
[5] E. Wu, S. Lawton, D. Olson, A. Rohrman, G. Kokotailo, *J. Phys. Chem.* **1979**, *83*, 2777-2781.
[6] R. Nakao, Y. Kubota, N. Katada, N. Nishiyama, K. Kunimori, K. Tomishige, *Appl. Catal. A* **2004**, *273*, 63-73.
[7] H. Yin, Y. Wada, T. Kitamura, S. Kambe, S. Murasawa, H. Mori, T. Sakata, S. Yanagida, *J. Mater. Chem.* **2001**, *11*, 1694-1703.

Author Contributions

D.Z. and P.K. conducted the catalytic tests and characterization measurements; D.Z., M.C., B.T., J.-D.G. und O.D. contributed to data evaluation and interpretation, as well as to writing of this paper.
